# AQSA—Algorithm for Automatic Quantification of Spheres Derived from Cancer Cells in Microfluidic Devices

**DOI:** 10.3390/jimaging10110295

**Published:** 2024-11-20

**Authors:** Ana Belén Peñaherrera-Pazmiño, Ramiro Fernando Isa-Jara, Elsa Hincapié-Arias, Silvia Gómez, Denise Belgorosky, Eduardo Imanol Agüero, Matías Tellado, Ana María Eiján, Betiana Lerner, Maximiliano Pérez

**Affiliations:** 1 Centro de Investigación Biomédica (CENBIO), Facultad de Ciencias de la Salud Eugenio Espejo, Universidad UTE, Quito 170527, Ecuador; ana.penaherrera@ute.edu.ec; 2 Special Coatings and Nanostructures Engineering (IREN), National Technological University, Buenos Aires 1706, Argentina; ramiro.isa@espoch.edu.ec (R.F.I.-J.); belerner@fiu.edu (B.L.); 3 Facultad de Informática y Electrónica Escuela Superior Politécnica de Chimborazo (ESPOCH), Riobamba 060104, Ecuador; 4 Facultad de Medicina, Universidad de Buenos Aires, Instituto de Oncología Ángel H. Roffo, Buenos Aires C1417, Argentina; ehincapie@institutoroffo.uba.ar (E.H.-A.); sgomezlopez@institutoroffo.uba.ar (S.G.); dbelgorosky@institutoroffo.uba.ar (D.B.); agueroimanol@gmail.com (E.I.A.); aeijan@institutoroffo.uba.ar (A.M.E.); 5 National Council for Scientific and Technical Research (CONICET), Buenos Aires C1414, Argentina; 6 Scientific and Technological Promotion National Agency, Buenos Aires C1425, Argentina; 7 VetOncologia Cancer Clinic Buenos Aires, Buenos Aires C1408, Argentina; vetoncologia@gmail.com; 8 Collaborative Research Institute Intelligent Oncology (CRIION), Hermann-Herder-Straße 4, 79104 Freiburg im Breisgau, Germany; 9 Department of Electrical and Computer Engineering, Florida International University, Miami, FL 33174, USA

**Keywords:** artificial intelligence, cancer, CSCs, algorithm, microfluidics, prediction

## Abstract

Sphere formation assay is an accepted cancer stem cell (CSC) enrichment method. CSCs play a crucial role in chemoresistance and cancer recurrence. Therefore, CSC growth is studied in plates and microdevices to develop prediction chemotherapy assays in cancer. As counting spheres cultured in devices is laborious, time-consuming, and operator-dependent, a computational program called the Automatic Quantification of Spheres Algorithm (ASQA) that detects, identifies, counts, and measures spheres automatically was developed. The algorithm and manual counts were compared, and there was no statistically significant difference (*p* = 0.167). The performance of the AQSA is better when the input image has a uniform background, whereas, with a nonuniform background, artifacts can be interpreted as spheres according to image characteristics. The areas of spheres derived from LN229 cells and CSCs from primary cultures were measured. For images with one sphere, area measurements obtained with the AQSA and SpheroidJ were compared, and there was no statistically significant difference between them (*p* = 0.173). Notably, the AQSA detects more than one sphere, compared to other approaches available in the literature, and computes the sphere area automatically, which enables the observation of treatment response in the sphere derived from the human glioblastoma LN229 cell line. In addition, the algorithm identifies spheres with numbers to identify each one over time. The AQSA analyzes many images in 0.3 s per image with a low computational cost, enabling laboratories from developing countries to perform sphere counts and area measurements without needing a powerful computer. Consequently, it can be a useful tool for automated CSC quantification from cancer cell lines, and it can be adjusted to quantify CSCs from primary culture cells. CSC-derived sphere detection is highly relevant as it avoids expensive treatments and unnecessary toxicity.

## 1. Introduction

Cancer stem cells (CSCs) belong to a subpopulation of undifferentiated cells present within tumors that have the potential for regeneration, differentiation, the maintenance of pluripotency, drug resistance, and tumorigenicity when transplanted into an innate host [1]. CSCs play a crucial role in chemoresistance and cancer recurrence. Thus, there is intense research that aims to understand CSC behavior. The importance of studying CSCs is that it contributes to the development of targeted therapies that are specifically directed to these cells, which may be more effective than traditional therapies that target all cells within the tumor [2].

In stem cell research, the main objective is to isolate as pure a population of stem cells as possible [3]. In the pursuit of this aim, sphere formation assay is accepted as a method for selecting and enriching CSCs [4]. Through anchorage-independent sphere culture with serum-free, nonadherent, and nutritionally deficient conditions, the differentiated tumor cells undergo apoptosis, while CSCs survive, adapt, and increase rapidly [5,6]. Indeed, the sphere culture approach represents an optimal method for enriching CSC subpopulations from whole tumors [6]. For instance, glioblastoma (GBM) is the most common malignant brain tumor in adults [7], and temozolomide (TMZ) has been utilized as the standard chemotherapy for newly diagnosed GBM since its initial Food and Drug Administration (FDA) approval in 2005. However, the widespread exposure to TMZ and highly heterogeneous and mutation-prone nature of GBM are responsible for these tumors to develop resistance to TMZ, which is driven mainly by a unique population of undifferentiated and highly tumorigenic cancer stem cells known as glioma stem cells (GSCs) [7].

Microfluidic technology is a powerful tool for stem cell isolation and characterization, considering also CSCs [8]. Microfluidic devices enable the observation of cells under specific conditions and durations. This precision is crucial for understanding cell behaviors and interactions in detail. Notably, microfluidic platforms allow for real-time on-chip analysis [8,9].

Artificial intelligence (AI), using machine learning, has an important role in developing new technologies for smart apps [10]. This is based on algorithm development that can iterate, learn, and improve. In particular, it is relevant for healthcare systems.

For example, some algorithms enable incidence and patient relapse prediction for chronic kidney disease [11]. In addition, in a COVID-19 context, AI development and synergy have been focused on blocking technologies for contaminant diagnosis to block pandemic effects [10]. In cancer, CSC identification allows the prediction of the treatment response, which is highly relevant to avoid expensive treatments and unnecessary toxicity.

Counting spheres and measuring their area is laborious, time-consuming, and operator-dependent. Therefore, sphere segmentation has been addressed by several works such as Tumor Spheres Quantification with Smoothed Euclidean Distance Transform [12] and others that are available in the literature in the form of Fiji macros like INSIDIA [13], Matlab packages [14], or standalone programs like SpheroidJ [15]. However, SpheroidJ is specialized in images with one sphere. To tackle this drawback, we propose software that runs the Automatic Quantification of Spheres Algorithm (AQSA), which is user-friendly and can be utilized with images with multiple spheres. Furthermore, with the idea of developing software that works in therapeutic prediction studies from tumor biopsies, this work concerns the analysis of spheres derived from tumor primary cultures.

The aim of this work was to develop a computational program that identifies spheres, counts them, and measures sphere area automatically with low computational cost in order to enable laboratories from developing countries to automatize sphere image analysis in a reproducible manner. The AQSA has a user interface for querying the specifications with which the images were obtained such as the image format, the magnification used to adjust the calculation of the area according to the scale, the folder where the images are saved, and the folder where the user wants to store the results. In this study, 10×-images of CSCs from the U251 human glioblastoma cell line cultured in a 12-multiwell plate were utilized to quantify the number of spheres in each image. In addition, 40×-images of LN229 human glioblastoma cell line were acquired when three different treatments were applied to develop predicting chemotherapy assays in cancer. Furthermore, images of spheres derived from primary cultures grown in microfluidic devices were analyzed to evaluate the software performance.

## 2. Materials and Methods

### 2.1. Sphere Formation Culture (3D)

Human GBM cell lines, LN229 and U251, were seeded in serum-free 1:1 mixture of Dulbecco’s Modified Eagle Medium and Ham’s F-12 nutrient mixture (DMEM F-12), under low-attachment and high-dilution (1000 cells·mL^−1^) conditions, with 0.2% B-27 (Gibco™ 17504-044, Grand Island, NY, USA), 20 ng·mL^−1^ recombinant human fibroblast growth factor (rhFGF) (R&D systems Minneapolis, MN, USA), and 20 ng·mL^−1^ recombinant human epidermal growth factor (rhEGF) (Sigma Aldrich, Co., St. Louis, MO, USA). After a week of sphere growth, the cultures were treated for 7 days with the conventional chemotherapy (temozolomide, TMZ) (LN229 70 µM; U251 50 µM), and with an iNOS inhibitor (S-methylisothiourea, SMT) (50 µM).

Images from three independent experiments were evaluated to optimize the algorithm.

Murine GBM cell line, GL26, was seeded in serum-free DMEM F12, under low-attachment and high-dilution (1000 cells·mL^−1^) conditions, with 0.2% B-27 (Gibco™ 17504-044, Grand Island, NY, USA), 10 µg·mL^−1^ recombinant human epidermal fibroblast growth factor (rhFGF) (R&D systems Minneapolis, MN, USA), and 10 µg·mL^−1^ recombinant human epidermal growth factor (rhEGF) (Sigma Aldrich, Co., St. Louis, MO, USA).

### 2.2. Primary Cultures in Microfluidic Devices

Nasal carcinoma and thyroid tumor samples from veterinary patients were obtained after surgery. They were stored in sterile disposable containers with 10 mL of DMEM-F12 supplemented with 2× antibiotic and antimycotic solution Gibco™ 15240062, Jenks, OK, USA. To process samples, fat tissue and necrotic regions were removed with sterile scissors. Afterwards, isolated tumoral tissue was placed in a Petri dish with 100 µL Anti-Anti (Gibco™ 15240062) to avoid contaminations and 5 mL of DMEM-F12 without serum were added. Small pieces were passed through a cell strainer (JetBiofil^®^ CSS013070, Shaanxi, China) to dissociate tumoral cells. Once a cell suspension was obtained, it was centrifuged in a 15 mL conical tube during 10 min at 400 G. Supernatant was discarded and pellet was resuspended in 2 mL of DMEM-F12. Cells were counted and concentration was determined with Luna-ll Automatic cell counter (Labtech, Mytogen House,11 Browning Road, Heathfield, East Sussex, UK).

Then, 5 mL of medium to form spheres was prepared (5 mL of DMEM-F12, 10 µL of rhEGF, 10 µL of rhFGF, 100 µL of B-27™, and 50 µL of Anti-Anti Gibco™ 15240062, Jenks, OK, USA) to resuspend cells and obtain a solution with the following concentrations to seed in the sterile microfluidic device: 1.5 × 10^5^, 3 × 10^5^, and 6 × 10^5^ cell·mL^−1^.

### 2.3. Microfluidic Device Fabrication

**Figure 1:**
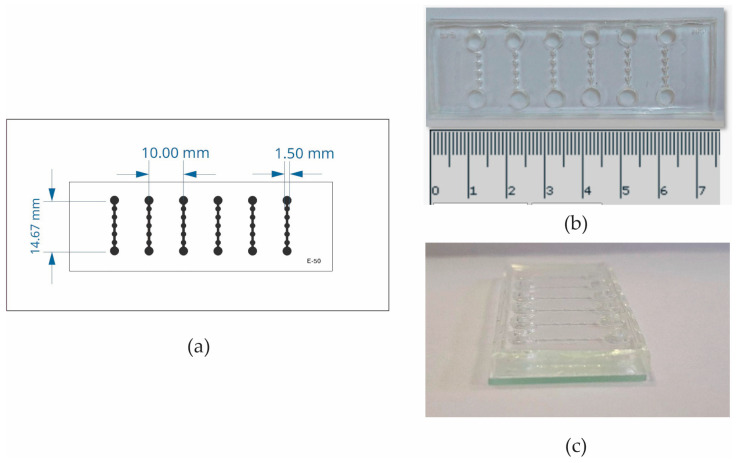
Microfluidic device architecture. (**a**) The microfluidic device design comprises 6 channels. Each channel has an inlet, 5 chambers, and an outlet. (**b**) Microfluidic device top view with scale in cm. (**c**) Microfluidic device side view.

Figure 1 presents the layout of the microfluidic chip which is formed by 6 channels provided with an inlet and an outlet. Each microchannel contains 5 chambers whose size fits in the visual field shown by 4× objective.

The channel layout for the microfluidic chip was designed using Layout Editor (free version Build 25 September 2023) software and transferred to a thermal imaging layer (TIL) with a 2400 pixels per inch (PPI) infrared source, as described by Olmos et al. 2020 [16]. Briefly, TIL was laminated onto a photopolymer plate that was exposed to UVA at 0.45 J at the back during 10 s, a part of the photopolymer was covered with a mask plate at the back, and the photopolymer plate was exposed to 0.45 J UVA light at the back for 20 s. Afterwards, the front part was exposed to UVA light at 19 J for 360 s. Then, the TIL was removed and the plate was washed with PROSOL N-1 solvent (supplied by Eastman Kodak, 343 State Street Rochester, NY 14650, USA) at 360 mm̭·min^−1^ before being dried in an oven for 30 min at 50 °C. The last exposure to UVC light at 10 J was for 17 min and to UVA light at 4 J for 2 min. Once the photopolymer female mold (Fmold) was ready, a mixture of epoxy resin and curing agent (Crystal-Tack, Novachem, Villa Martelli, Argentina) was poured onto the Fmold to replicate the design and obtain a male mold. Subsequently, a mixture of PDMS and curing agent in a 10:1 weight ratio (Sylgard 184 silicone elastomer kit, Dow Corning, Midland, MI, USA) was poured onto the epoxy resin mold and cured in an oven at 40 °C overnight.

### 2.4. Image Acquisition by Microscopy

Phase-contrast microscope (Nikon eclipse TE2000-S, Nikon corporation, Tokyo, Japan) and its software NIS-Elements BR 2.30 were utilized to acquire images of LN229 and U251 human glioblastoma cell lines. A hundred 10×-images of U251 were used to quantify sphere number manually and compare it to the sphere number reported by AQSA.

With the idea of programming the algorithm to evaluate response to chemotherapy treatment, cultures of spheres from the LN229 line were used. Thus, 40×-images of LN229 human glioblastoma cell line were acquired at day 12 (objective: LD A-Plan 40×/0.55 Ph1). Then, the algorithm was programmed to quantify number of spheres and to calculate the area in square micrometers in control wells and in threatened TMZ and SMT.

Tumors are complex entities made up of tumor cells and other cells of the tumor microenvironment; therefore, the algorithm must consider these differences in tumor biopsies. The growth of spheres was evaluated from primary cultures of canine patients. The idea of the experiment is not only to analyze growth from tumor biopsies but also to follow the growth of individual spheres for which the cultures were grown in the microdevice shown in Figure 1. Sphere images were acquired using an inverted Zeiss microscope Axio Vert (Carl Zeiss, Suzhou, China) A1 (objective: 10×/0.25 Ph1).

To analyze the influence of different microscopes on the results, images of spheres derived from murine glioblastoma cell line GL26 at seven days of growth were acquired with two microscopes, a phase-contrast microscope (Nikon eclipse TE2000-S) (objective: Nikon Plan Fluor10×/0.30 Ph1 DL) and an inverted Zeiss microscope (Axio Vert. A1) (objective: 10×/0.25 Ph1).

### 2.5. Cell Number and Reported Area Experiment

A known concentration of LN229 cells was cultured, and images of derived spheres were acquired. The area occupied by formed spheres was determined by the AQSA algorithm and the relation between the number of cells, and the area was determined by a simple linear regression with Graph Pad Prism9 software.

### 2.6. Software Development

**Figure 2:**
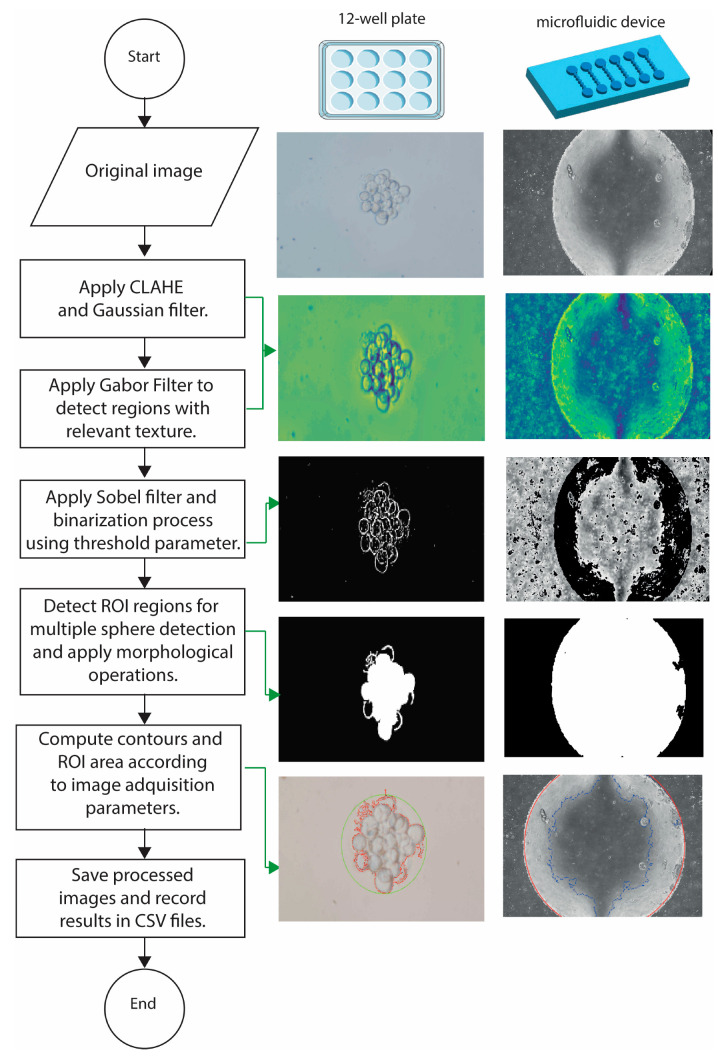
Flow diagram of image analysis process. Schematic representation of AQSA performance in uniform (12-multiwell plate) and nonuniform (microfluidic chip) environments.

A graphical user interface (GUI) has been developed called Spheres Interface (Figure S1) to run AQSA—Automatic Quantification of Spheres Algorithm using Python. It is mainly based on PySimpleGUi, SKIMAGE, and MATPLOTLIB libraries to detect and plot regions with spheres over images. First, an interphase is displayed to ask the researcher about the magnification lens utilized to acquire images. Once the lens has been specified, the program applies the appropriate scale to quantify the sphere number and area in micrometers. Briefly, the contrast-limited adaptive histogram equalization (CLAHE) and Gaussian filters are used to homogenize the image contrast and reduce noise. Then, a binarization process using an adaptive threshold is performed. Afterwards, the region of interest (ROI) is identified according to image resolution. Once ROI is detected, the Sobel filter is applied to detect borders of spheres in ROIs, and morphological operations are performed to improve object detection. Finally, the percentage of the area used by each sphere in ROI is computed and the processed image and a comma-separated value (CSV) file with analysis results are saved at the user-chosen location [17]. This algorithm has been evaluated with images that present different environments such as uniform (12-multiwell plate) and nonuniform backgrounds (microfluidic device), as described in Figure 2. The advantage of the AQSA algorithm is its robustness in determining the area of a sphere culture at different resolutions including from the view of a well. This software is accessible from a GitHub repository [18].

#### 2.6.1. Image Preprocessing

**Figure 3:**
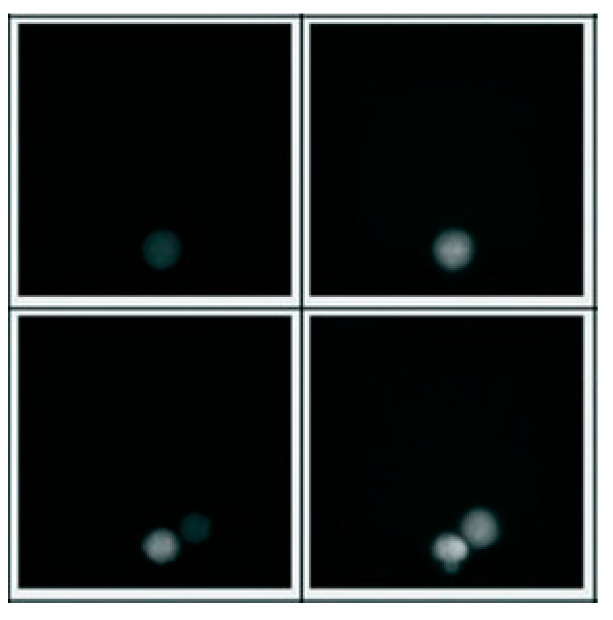
CLAHE and Gaussian filtering effect. Image quality improvement applying CLAHE and Gaussian filter.

AQSA has a preprocessing stage which is an important step in image analysis. CLAHE [19] and the Gaussian filter for smoothing were used to improve the image quality. Figure 3 presents the results of the CLAHE and Gaussian filtering during the preprocessing stage.

#### 2.6.2. Gabor Filter Bank

The Gabor filter has real and imaginary components representing orthogonal directions. In two dimensions (2D), Gabor filter is defined as shown in Equation (1) [20]:

(1) $$g\left(x,\;y\right)=\frac{f^{2}}{\pi \gamma \eta }\exp-\left[\frac{x^{\prime 2}f^{2}}{\gamma^{2}}+\frac{y^{\prime 2}f^{2}}{\eta^{2}}\right]e^{-j2\pi fx^{\prime }}\;\;\;\;\;$$

where *x*′ = *x* cos *θ* + *y* sin *θ*, *y*′ = −*x* sin *θ* + *y* cos *θ*. Furthermore *x*, *y* are the coordinates of frequencies in the reference system, *x*′, *y*′ in *θ* orientation of Gabor function, *f* represents the distance from origin to the center of Gaussian function, and *γ* and *η* characterize the sharpness of Gaussian function along the major and minor axes.

**Figure 4:**
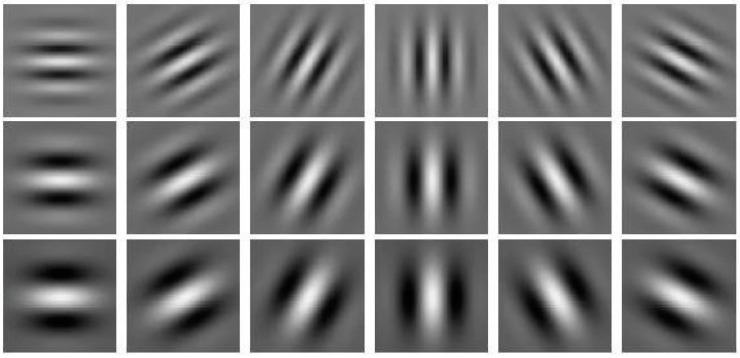
Gabor filter set application. Parameters of Gabor filter set: *f* = [1/4, 1/6, 1/8], *size* = 21, 6 rotations: *θ* = [$0^{o},\;30^{o},\;60^{o},\;90^{o},\;120^{o},\;150^{o}$], and *γ* = 10, η = 0.5.

A set of Gabor filters is used to detect features in a given input image. This set can be computed in a space of M scales (frequencies) and N rotations to ensure invariance. The real component of this set is presented in Figure 4, where AQSA uses a set composed of 16 filters with 3 scale values and 6 different angles for rotation values. This set avoids feature redundancy and optimizes the processing time. 

#### 2.6.3. Sobel Filter

This computes the gradient of the image intensity in each pixel. The operator calculates the magnitude of the largest possible change, the direction, and the sense from dark to light. Mathematically, the operator uses two 3 × 3 kernels to convolve with the original image and calculate approximations to the horizontal and vertical derivatives. The process is presented in Equations (2) and (3) [21].

(2) $$G_{x}=\left[\begin{array}{ccc} -1 & 0 & +1\\ -2 & 0 & +2\\ -1 & 0 & +1 \end{array}\right]\;\;*A\;\;\;\;\;\;\;\;\;\;\;\;\;\;\;\;\;\;\;\;\;\;G_{y}=\left[\begin{array}{ccc} -1 & -2 & -1\\ \;\;\;0 & \;\;\;0 & \;\;\;0\\ +1 & +2 & +1 \end{array}\right]\;\;*A\;\;$$

where $G_{x}$, $G_{y}$ are the gradient in horizontal and vertical directions, and A is the image.

(3) $$\;\;G=\sqrt{G_{x}^{2}+G_{y}^{2}}\;\;\;\;\;\;\;\;\;\;\;\;\;\;\;\;\;\;\;\;\;\;\;\;\;\;\;\;\;\;\;\;\;\;\;\;\;\;\;\;\;\;\theta ={\tan^{-1}}^{2}\left(G_{x},\;G_{y}\right)\;\;$$

where G represents the gradient magnitude and θ is the gradient direction.

#### 2.6.4. Binarization Method and Morphological Operations

Using the Otsu method, the threshold value for binarization is calculated for sphere regions. The threshold value for the binary function is computed according to the image histogram [22].

Finally, the closing and dilate morphological operations are used to enlarge the boundaries of foreground regions and shrink background color holes in an image using a structuring element [23].

### 2.7. Software Validation

A known number of cells was cultured in the 12-multiwell plate, and images of spheres derived from different numbers of cells were analyzed to assess the correlation between the AQSA algorithm reported area and the number of cells.

In addition, a hundred images acquired from U251 cells were processed with the algorithm and the sphere number reported was compared with the manual count. Automatic area results were compared with manual annotations and with an open-source software Fiji (ImageJ 1.54f) [24], which is a Java-based software with several plugins that facilitate scientific image analysis based on a semiautomatic pipeline consisting of (i) conversion of red, green, and blue (RGB) image into grayscale; (ii) manual intensity thresholding; (iii) hole filling; and (iv) small particle removal. The 47 images of the dataset were used to validate the performance of the AQSA algorithm in segmenting sphere borders concerning a manual operator. The operator drew each sphere border to assess area detection. A comparison between areas measured from masks drawn by a manual operator and areas provided by AQSA is carried out to evaluate the algorithm’s performance.

### 2.8. Sphere Formation Efficiency Calculation

The sphere formation efficiency (SFE) indicates the percentage of cells within a culture that are capable of forming a sphere from a single cell [25,26]. It is calculated by applying the following equation:

(4) $$SFE\%=\frac{number\;of\;spheres}{number\;of\;cell\;sseeded}\times 100$$

### 2.9. Statistical Analysis

Statistical analyses were performed using Graph Pad Prism9 software. Experimental values for continuous variables are expressed as the mean standard error of the mean, and the Student’s *t*-test was used as appropriate to evaluate the significance of differences in data between groups.

## 3. Results

Automatic image processing encompasses sphere identification, counting, and area measurement. The assessment of this process includes (i) determining the correlation between the known cultured number of cells and the area reported by the algorithm, (ii) a comparison between sphere manual count and algorithm quantification, and (iii) software validation in the 12-multiwell plate and in microfluidic devices.

### 3.1. Automatic Image Processing Assessment

#### 3.1.1. Determining Correlation Between Known Cultured Number of Cells and Area Reported by the Algorithm

**Figure 5:**
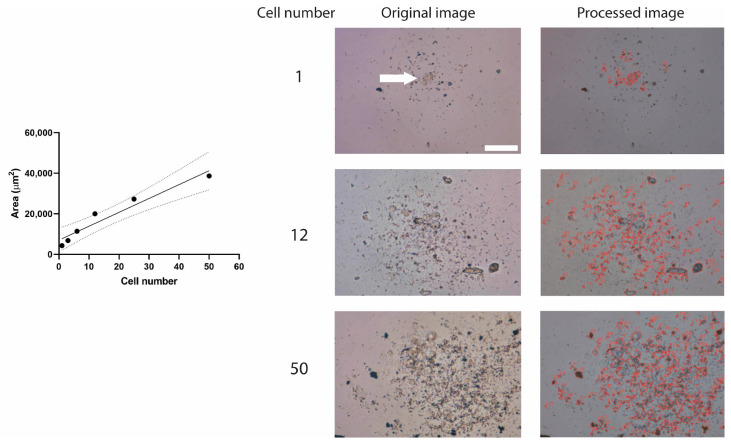
Relation between average area and cell number. Representative 40× images of LN229 cell line. White arrow indicates cell position. Scale bar: 100 μm. Solid line represents linear regression line. While dashed lines are the boundaries of all possible straight lines.

It was observed that the algorithm performance is better when images present a uniform background. When images have a nonuniform background, artifacts are interpreted as spheres. Accordingly, a similar contrast among images is desired to improve sphere detection. A known number of cells was cultured, and it was determined that there is a direct relation (R^2^ = 0.9357) between the area reported by the AQSA algorithm and the number of cultured cells (from 1 to 50 cells), as presented in Figure 5. Images obtained at cell number 3, 6, and 25 are presented in Figure S2.

#### 3.1.2. Comparison Between Sphere Manual Count and Algorithm Quantification

**Figure 6:**
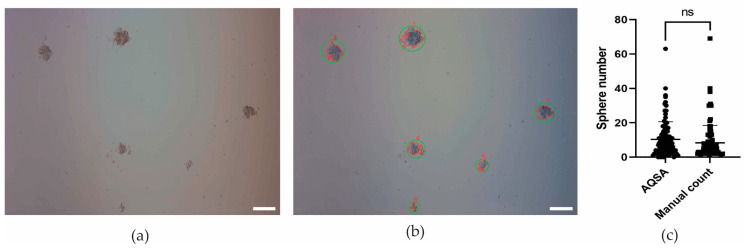
Sphere detection, identification, and quantification in 12-well plate. (**a**) Original 10× image. (**b**) Detected spheres are circled in green and numbered in red. U251 human glioblastoma cell line. Scale bar 200 μm. (**c**) Sphere number comparison between AQSA algorithm and manual count performed with a hundred 10× U251 human glioblastoma cell line images. Student’s *t*-test (*p* = 0.167); ns means no statistically significant difference (*p* > 0.05).

Regarding spheres quantification, algorithm performance was compared to manual count with the mean difference between the two methods, and the standard deviation of the difference was estimated (mean ± SD: 2 ± 4, ns by Student’s *t*-test) (Figure 6c). Also, the algorithm allows sphere identification with numbers, as illustrated in Figure 6a,b, which enables the tracking of individual spheres in time.

#### 3.1.3. Software Validation Results

Several approaches for the automatic segmentation of spheroid images exist in the literature. Recently, a set of open-source tools for spheroid segmentation called SpheroidJ [15] was published. However, the authors use deep learning to generate the method, which requires a large image dataset with the spheroids they are identified. This process demands a high computational cost during model training. In addition, it needs images with similar contrast and can be applied to images with just one sphere. To overcome these limitations, the AQSA algorithm applies the Gabor filter at the image preprocessing stage to obtain the regions with high response to texture segmentation. Afterward, the Sobel filter is suitable for processing features from images filtered using Gabor space [27,28]. Consequently, these tools allow the generalization of image processing with distinct contrast features in different scenarios in a short time and with a low computational cost.

### 3.2. Software Application to Determine Sphere Area from Cells Cultured in 12-Multiwell Plate

**Figure 7:**
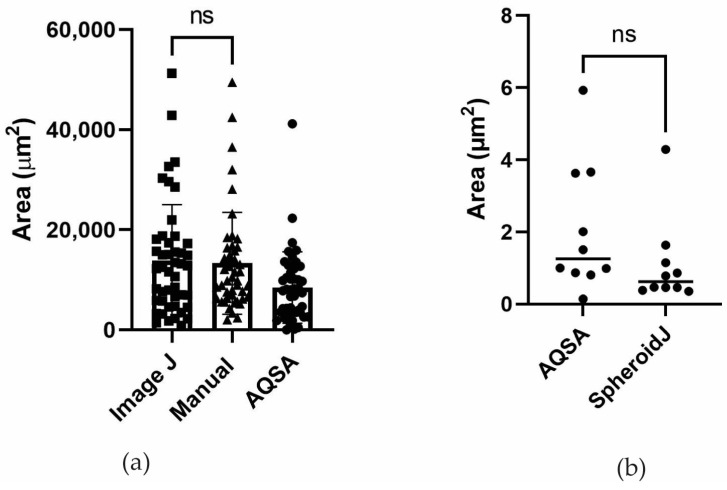
Area quantification. (**a**) Area measurement comparison among ImageJ, manual, and AQSA for images with more than one sphere. (**b**) Area measurement comparison between AQSA and SpheroidJ for images with one sphere (Student’s *t*-test (*p* = 0.173); ns means no statistically significant difference (*p* > 0.05)).

The AQSA area measurement method was tested on 47 images of the LN229 cell line (Figure 7). Automatic area results were compared with manual annotations and with open-source software Fiji (ImageJ 1.54f) [16], which is a Java-based software with several plugins that facilitates scientific image analysis based on a semiautomatic pipeline consisting of (i) conversion of RGB image into grayscale, (ii) manual intensity thresholding, (iii) hole filling, and (iv) small particle removal. The 47 images of the dataset were used to validate the performance of the AQSA algorithm in segmenting sphere borders related to a manual operator. The operator drew each sphere border to assess area detection. A comparison of the mean area measured from masks drawn by a manual operator and areas provided by AQSA is computed to evaluate the algorithm’s performance. As reported in Figure 7a, the mean sphere area measurements performed by a manual operator and a command sequence in ImageJ are not significantly different. The mean sphere area measurement performed by the AQSA algorithm is slightly lower. This difference could be attributed to the nonuniform contrast of images. However, the time to perform the sphere area measurement of each image is 0.3 s. This is significantly less than the time needed to perform the sphere mean area measurement of each manual image processing (by drawing the contour of the spheres, approximately 5 min per image). This time optimization allows us to perform the image analysis in a reproducible manner and to analyze the large number of images, avoiding human errors. Shown in Figure 7b, a comparison between AQSA and SpheroidJ area measurements was performed, and it was determined that there is no statistically significant difference.

### 3.3. Sphere Algorithm Enables Measurement of Treatment Response

**Figure 8:**
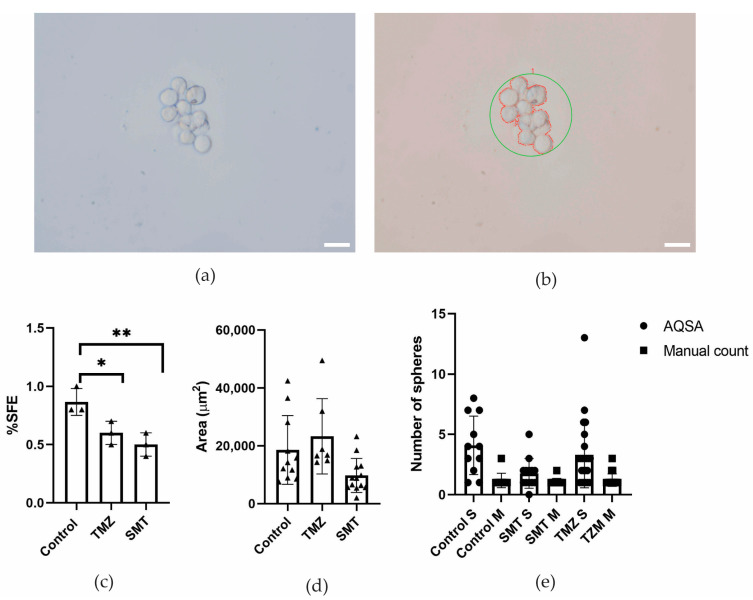
Treatment response. Upper panel shows an image of LN229 cell-line-derived sphere. (**a**) Original 40× image and (**b**) AQSA analyzed image. Lower panel indicates treatment response according to (**c**) sphere formation efficiency (SFE), (**d**) sphere area, and (**e**) sphere number. Scale bar corresponds to 50 μm (* *p* ≤ 0.05 and ** *p* ≤ 0.01).

LN229 cell line-derived spheres were exposed to TMZ and SMT treatments. Figure 8b shows a single derived sphere detected by the AQSA algorithm with the measured area indicated by a red contour; the detected sphere is circled in green and is identified as sphere 1. According to area measurements, it was possible to observe a treatment response. TMZ treatment does not reduce sphere area (Figure 8d). This finding is in accordance with the literature. Chemotherapy produces an evolutionary selective pressure resulting in the expansion of drug-resistant GSCs [25]. Interestingly, the area measurements reported by the AQSA algorithm showed that SMT treatment generates an effect to reduce the sphere area. This finding agrees with sphere formation efficiency percentage, as SMT significantly decreases SFE percentage (Figure 8c) in comparison to control (*p* ≤ 0.01).

Regarding sphere number, the manual count shows a smaller number of spheres than the sphere number reported by the AQSA algorithm. This can be attributed to artifacts such as crystals or cell debris that have been recognized as spheres.

### 3.4. Software Application in Spheres Cultured in Microfluidic Devices

**Figure 9:**
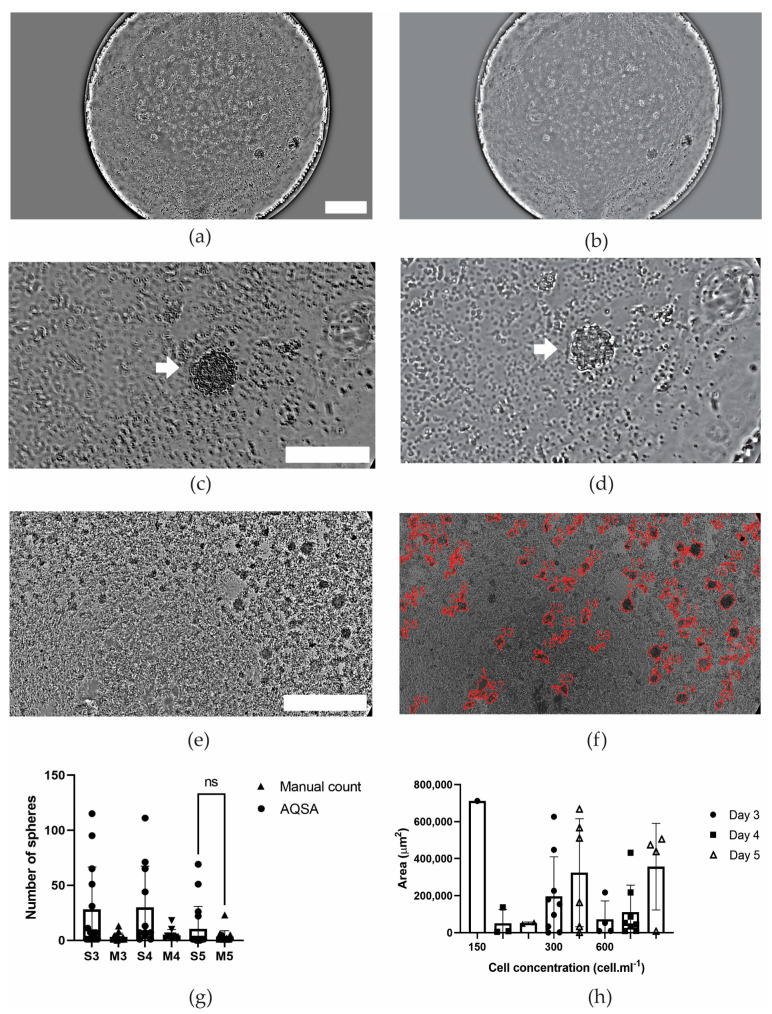
High-grade compact solid canine thyroid carcinoma. (**a**) 4× image of a tracked in time sphere inside a microfluidic device chamber at day 4. Scale 100 um. (**b**) 4× image of a tracked in time sphere inside a microfluidic device chamber at day 5. (**c**) 40× image of sphere tracking at day 4. (**d**) 40× image of sphere tracking at day 5. Scale bar 100 µm. (**e**) Original 10× image of sphere derived from thyroid cancer cells. (**f**) Detected spheres reported by AQSA. Scale bar 100 µm. (**g**) Sphere manual count compared to AQSA count at days 3–5. (**h**) Area measurement by AQSA at different cell concentrations at days 3–5. White arrows indicate the tracking of the same sphere during time and ns means no statistically significant difference (*p* > 0.05).

**Figure 10:**
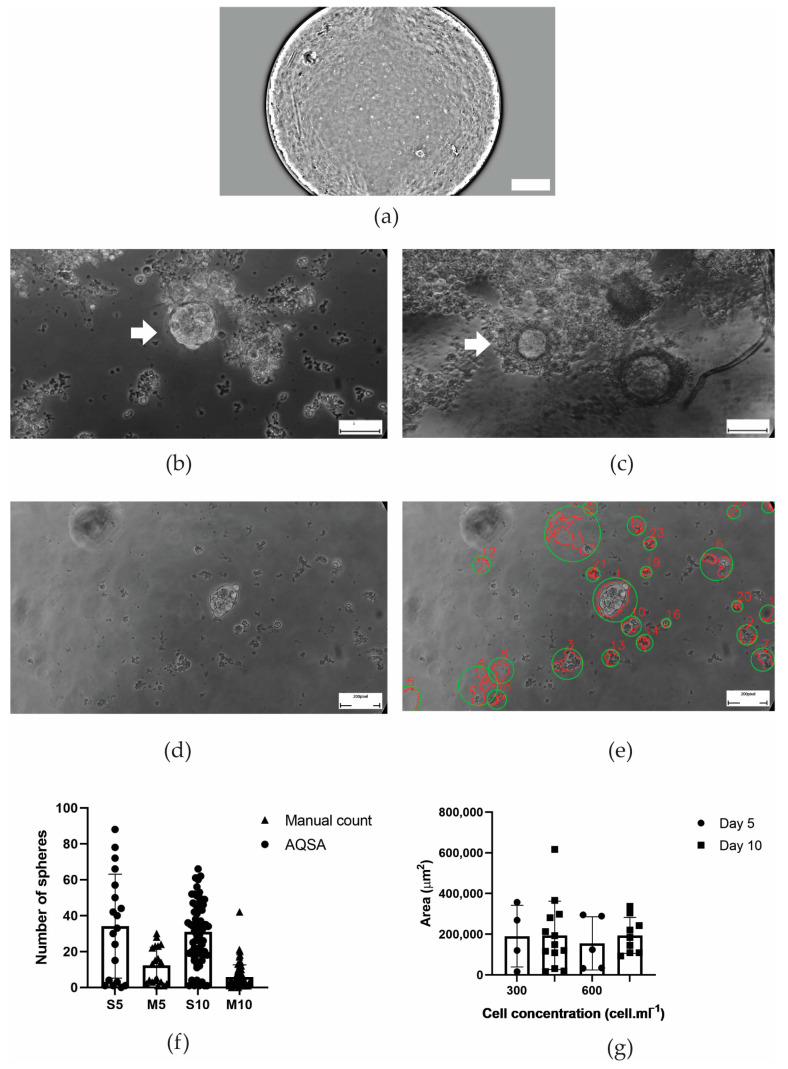
Pleomorphic neoplastic lesion with carcinomatous pattern in the canine nasal cavity. (**a**) 4× image of a tracked in time sphere inside a microfluidic device chamber on day 10. Scale: 100 µm. (**b**) 40× image of sphere tracking day 10. (**c**) 40× image of sphere tracking day 12. Scale bar 50 µm. (**d**) Original 10× image of sphere derived from nasal tumor cells. (**e**) Detected spheres reported by AQSA. Scale bar 100 µm. (**f**) Sphere manual count compared to AQSA count on day 5 and day 10. (**g**) Area measurement by AQSA. White arrows indicate the tracking of the same sphere during time, ns means no statistically significant difference (*p* > 0.05), and green circles mark the counted spheres.

Image analysis of primary culture cells in microfluidic devices is more challenging due to several factors such as nonuniform contrast, the presence of red blood cells (erythrocytes), artifacts considered spheres, as shown in Figure S3, and artifacts outside the microfluidic chamber. In addition, according to the type of tumor, spheres might be agglomerated. This agglomeration complicates separation and detection. Therefore, a refinement will be made to improve sphere recognition in the next version of the algorithm specialized in primary culture cells. Two tumor samples from two veterinary patients were processed. Therefore, two types of primary tumor cells were cultured in microfluidic devices: tumor cells from high-grade compact solid canine thyroid carcinoma (Figure 9), and pleomorphic neoplastic lesions with carcinomatous patterns in the canine nasal cavity cells (Figure 10).

In the case of thyroid carcinoma, spheres formed on day 3, and the same sphere could be tracked in the time course (Figure 9a–d) due to the microfluidic chamber where it was located being identified. Sphere detection by the AQSA algorithm was difficult, as primary cultures present erythrocyte groups that resemble spheres (Figure 9e,f). In addition, the shadow of the microfluidic chamber border produces a nonuniform background in the image. However, the manual count and the number of spheres reported by the proposed method were not significantly different on day 5 (Figure 9g). This might be attributed to erythrocyte presence reduction at day 5, considering the medium renewal. Regarding area measurement, AQSA measurements presented an increase in area for 300 and 600 cell·mL^−1^ concentrations from day 3 to day 5 (Figure 9h).

In the case of nasal tumor, spheres formed on day 5, and the same sphere could be tracked in the time course (Figure 10a,b) due to the microfluidic chamber where it was located being identified. Sphere detection by the AQSA algorithm compromised cellular debris and erythrocytes that resemble spheres being present (Figure 10d,e). In addition, the microfluidic chamber wall is made of PDMS to replicate mold imperfections that can be recognized as spheres. Therefore, the manual count and the number of spheres reported by the AQSA algorithm were higher on day 5 and day 10 (Figure 10f). According to area measurements, the proposed method allowed the confirmation of an increase in areas for 300 and 600 cell·mL^−1^ concentrations from day 5 to day 10 (Figure 10g).

### 3.5. Comparison of AQSA Sphere Detection in Two Microscopes

**Figure 11:**
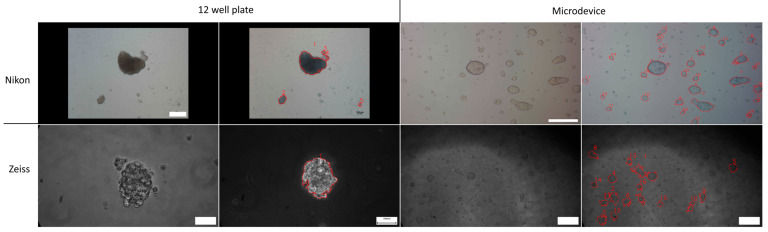
Comparison of AQSA sphere detection in two microscopes. Upper panel shows images acquired with phase-contrast Nikon microscope, and lower panel presents images obtained with an inverted Zeiss microscope. Upper panel 4× 12-well plate image scale bar corresponds to 500 µm, while 10× microdevice image scale bar corresponds to 200 µm. Lower panel 40× 12-well plate image scale bar corresponds to 50 µm, while 10× microdevice image scale bar corresponds to 100 µm.

Sphere images from murine GMB cell line GL26 were acquired with two microscopes to evaluate if different microscopes would influence results. As can be seen in Figure 11, AQSA sphere detection (red border) was precise in 12-well plates for Nikon and Zeiss microscopes. For spheres cultured in a microfluidic device, Nikon microscope images showed better results, as illumination is uniform. However, in a microfluidic device with Zeiss microscope images, several spheres were not detected due to different contrast. Therefore, AQSA detection is not compromised when using different microscopes when using a 12-well plate. In microfluidic devices, some illumination parameters have to be improved to avoid their influence on results.

## 4. Discussion

An automatic segmentation method for spheres derived from cancer cell detection was presented. This includes preprocessing, filtering, and morphological operations stages. Preprocessing and filtering are the main processes in AQSA for sphere detection. The CLAHE method was used to reduce the variation of contrast in images, and the Gabor and SOBEL filters were used for texture analysis and edge detection, respectively. The AQSA process includes CLAHE as a preprocessing step to eliminate different levels of image contrast, and interest objects can be differentiated. Furthermore, the Gabor filter bank detects the textures in images. This filter significantly improves the segmentation results, especially when the background is not uniform. These processes ensure that AQSA is robust enough to process images with distinct equipment, experimental characteristics, and environmental conditions. Therefore, this tool enables the tracking of sphere growth in real time through live cell imaging.

According to the results, the performance of the AQSA algorithm analyzes a large number of images in 0.3 s per image with a low computational cost, which enables developing countries to analyze sphere images in an efficient and reproducible manner without a powerful computer. AQSA performance is better when images present a uniform background, as in the case of images obtained from a 12-well plate. This was also observed using two microscopes (Nikon and Zeiss). In images with nonuniform backgrounds, such as images acquired from microfluidic devices, artifacts are probably interpreted as spheres. Nevertheless, sphere detection accuracy was better when the Nikon microscope was utilized to capture images of spheres cultured in microfluidic devices, as illumination was uniform. Moreover, the AQSA algorithm reported an area with a direct relation to the number of cultured cells. Furthermore, our algorithm identifies spheres with numbers, which makes it possible to track the same sphere in the same time. This algorithm measures the area of spheres in one or more of them automatically. Area measurements of images that present one sphere were compared between AQSA and SpheroidJ, and there was no statistically significant difference. The AQSA algorithm can detect multiple spheres in an image; this is an advantage in comparison with plugins that are designed to measure sphere area in images that have just one sphere. Indeed, this capacity-optimized time enabled the observation of treatment response in LN229 cell-line-derived spheres. Sphere area measurements showed that it decreases when SMT is applied. Therefore, this algorithm can be a useful tool for automated CSC quantification from cancer cell lines and can be adjusted to quantify CSC from primary culture cells, like in our experiments in microfluidic devices where the AQSA algorithm was applied. In the case of thyroid carcinoma, the detection of spheres was difficult due to the presence of erythrocytes. Nevertheless, the manual count and the value reported by the AQSA algorithm on day 5 were not significantly different, as medium renewal enables erythrocyte reduction. Furthermore, area measurement performed by AQSA shows an area increment at 300 cell·mL^−1^ and at 600 celḽ̭·mL^−1^ concentrations from day 3 to day 5.

With regard to nasal carcinoma, cellular debris and erythrocyte presence compromised sphere detection and sphere number count. However, the sphere area measurement performed by the proposed algorithm showed an area increase from day 5 to day 10. Despite the limitations inherent to primary culture, spheres cultured in devices that show uniform backgrounds can be efficiently detected, identified to be tracked in the time course, counted, and measured with the current version of the AQSA algorithm. With regard to machine learning methods, AQSA does not require an extensive database of images to segment spheres. Moreover, this method does not need an elevated computational cost, including the analysis of images with high resolution. However, AQSA could be the main step to classify different types of spheres using artificial neuronal networks (ANNs) and support vector machines (SVMs), among others, or to obtain the sphere patterns to describe specific characteristics through convolutional neuronal networks (CNNs). These methods could require manually labeling images from experts in many datasets. The main difference with other proposed methods is that AQSA can segment spheres in images with low resolution and at different zoom levels. Furthermore, the proposed algorithm is capable of identifying more than one sphere to count and compute their areas. The proposed approach will provide, in the future, automated quantitative analysis of sphere number and area. It can be combined with machine learning to perform automatic segmentation, enabling predictive associations between treatment and phenotypic changes. Future work is focused on improving results during the erythrocyte’s presence or nonuniform background using novel techniques such as machine learning algorithms for the detection and classification of spheres.

## Data Availability

Relevant data were uploaded with the name of the project: AQSA—algorithm for automatic quantification of spheres derived from cancer cells in microfluidic devices, at the following link: https://figshare.com/account/home#/projects/213385 (accessed on 16 July 2024). Images were uploaded at the following link: https://www.kaggle.com/datasets/ramiroisajara/spheres-cancer-celllines (accessed on 17 July 2024).

## Supplementary Materials

The following supporting information can be downloaded at: https://www.mdpi.com/article/10.3390/jimaging10110295/s1, Figure S1: Captions of sphere interphase. User-friendly interphase displayed during automatic image analysis. (a) One sphere. (b) More than one sphere; Figure S2: Relation between average area and cell number. Representative 40× images of LN229 cell line at cell number 3, 6, and 25. Scale bar: 100 µm; Figure S3: Sphere detection and identification in microfluidic chambers. (a) Original 10× image primary culture of nasal tumor cells in a microfluidic chamber and (b) AQSA algorithm sphere detection. Scale bar 100 µm.

## Nomenclature

Terms and definitions.

**Term**	**Definition**
B-27™	It is an optimized serum-free supplement used to support the low- or high-density growth.
Spheroid formation	It is a common assay used to measure the self-renewal and multipotent nature of the cancer stem cell subpopulations within a tumor or cancer cell line.
Computer vision	It is a field of computer science that focuses on allowing computers to identify and understand objects and people in images and videos.
Image processing	There are two main types of image processing: image filtering and image warping.
Image filtering	It changes the range (i.e., the pixel values) of an image, so the colors of the image are altered without changing the pixel positions.
Image warping	It changes the domain (i.e., the pixel positions) of an image, where points are mapped to other points without changing the colors.
Image segmentation	It is a commonly used technique in digital image processing and analysis to partition an image into multiple parts or regions, often based on the characteristics of the pixels in the image.
Image binarization	It is a process that converts the color or grayscale original captured images into digital binary images consisting of small black and white pixels.
ROI—region of interest	It is a part of an image that you want to filter or process in some way. An ROI can be represented as a binary mask.
Enhance Local Contrast	CLAHE implements the method contrast-limited adaptive histogram equalization for enhancing the local contrast of an image.
Edge detection	It includes a variety of mathematical methods that aim at identifying edges, defined as curves in a digital image at which the image brightness changes sharply or, more formally, has discontinuities.
Contours	It can be explained simply as a curve joining all the continuous points (along the boundary), having same color or intensity. The contours are a useful tool for shape analysis and object detection and recognition.
Object counting	It is a technique used in image processing to automatically detect and count the number of objects in an image. It involves an edge detection, segmentation, and feature extraction to find objects of interest in the image.
Feature extraction	It refers to the process of transforming raw data into numerical features that can be processed while preserving the information in the original dataset.
Machine Learning	The use and development of computer systems that are able to learn and adapt without following explicit instructions, by using algorithms and statistical models to analyze and draw inferences from patterns in data.
Deep Learning	A type of machine learning based on artificial neural networks in which multiple layers of processing are used to extract progressively higher level features from data.
Python	It is an interpreted, object-oriented, high-level programming language with dynamic semantics. Its high-level built in data structures, combined with dynamic typing and dynamic binding, make it very attractive for Rapid Application Development, as well as for use as a scripting or glue language to connect existing components together.
Plugin	It is a software component that adds a specific feature to an existing computer program.
Graphical User Interface	GUI is an interface between human and machine. The objective of this graphical interface is to represent the backend code of a system in the clearest way possible for the user to simplify daily tasks.
