# Supplementary material for: AQSA—Algorithm for Automatic Quantification of Spheres Derived from Cancer Cells in Microfluidic Devices
Source: J Imaging. 2024 Nov 20;10(11):295. doi: 10.3390/jimaging10110295 (PMC11595607; doi:10.3390/jimaging10110295)

Supplementary information.

Figure S1. Captions of Sphere interphase. User friendly interphase displayed during automatic image analysis. a) one sphere. b) More than one sphere.

a)

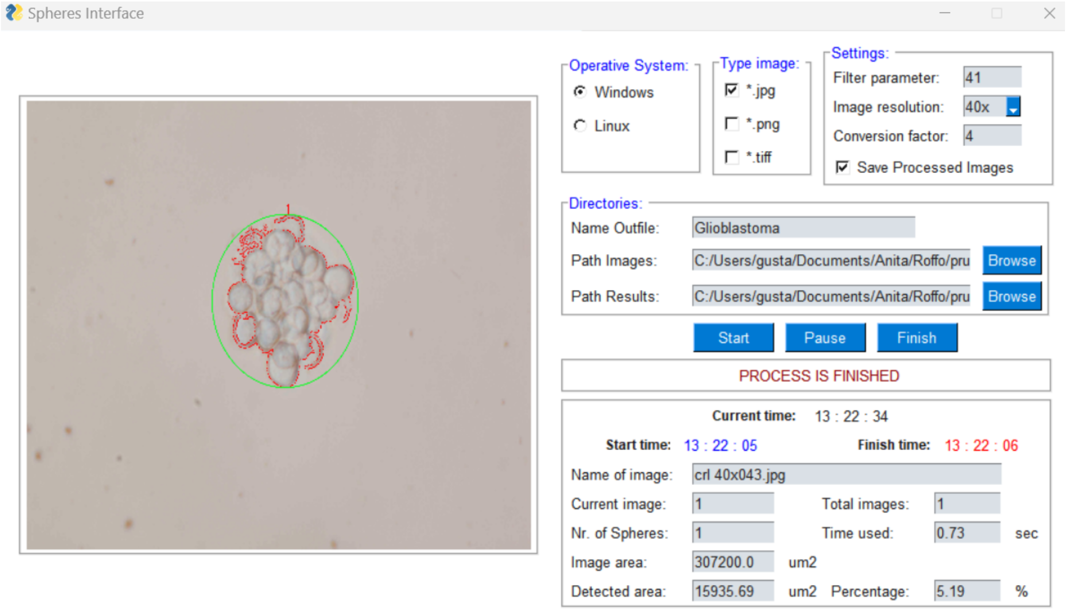

b)

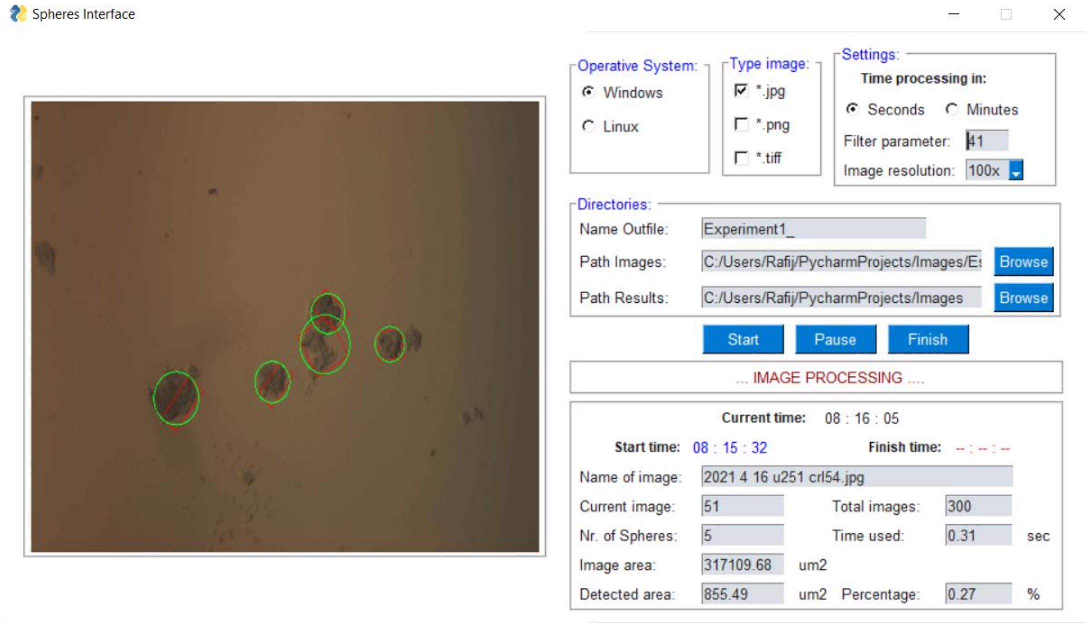

Figure S2. Relation between average area and cell number. Representative 40× images of LN229 cell line at cell number 3, 6 and 25. Scale bar: 100 µm

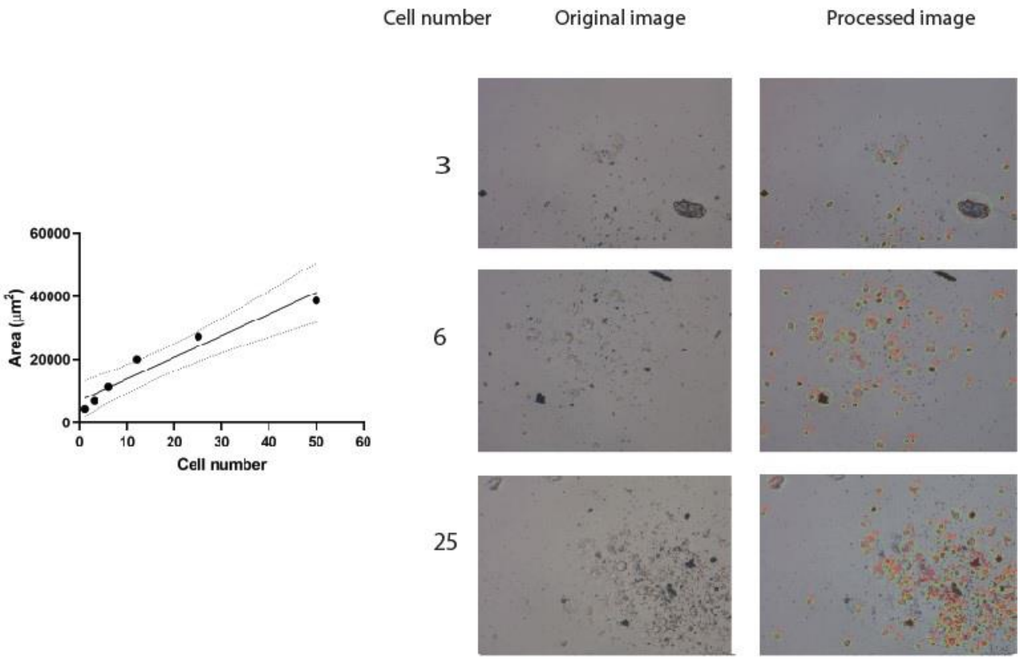

Figure S3. Sphere detection and identification in microfluidic chambers. a) Original 4× image primary culture of nasal tumor cells in a microfluidic chamber and b) AQSA algorithm sphere detection. Scale bar 100 µm.

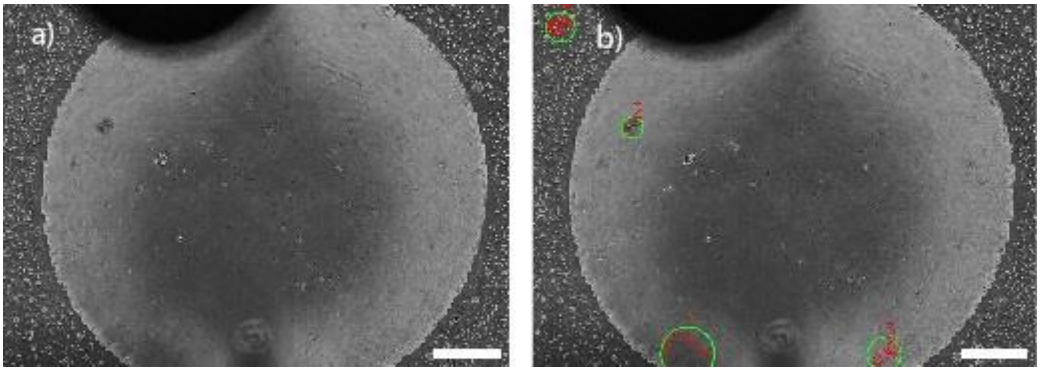

Supplement: Supplementary file 1 [file jimaging-10-00295-s001.zip › jimaging-3226026-supplementary.pdf]
